# Droplet-based microtumor model to assess cell-ECM interactions and drug resistance of gastric cancer cells

**DOI:** 10.1038/srep41541

**Published:** 2017-01-27

**Authors:** Minjeong Jang, Ilkyoo Koh, Seok Jae Lee, Jae-Ho Cheong, Pilnam Kim

**Affiliations:** 1 KAIST, Department of Bio and Brain Engineering, Daejeon 34141, Republic of Korea; 2 Department of Nano Bio Research, National NanoFab Center, Daejeon 34141, Republic of Korea; 3 Yonsei University College of Medicine, Department of Surgery, Seoul 03722, Republic of Korea

## Abstract

Gastric cancer (GC) is a common aggressive malignant tumor with high incidence and mortality worldwide. GC is classified into intestinal and diffuse types according to the histo-morphological features. Because of distinctly different clinico-pathological features, new cancer therapy strategies and *in vitro* preclinical models for the two pathological variants of GC is necessary. Since extracellular matrix (ECM) influence the biological behavior of tumor cells, we hypothesized that GC might be more similarly modeled in 3D with matrix rather than in 2D. Herein, we developed a microfluidic-based a three-dimensional (3D) *in vitro* gastric cancer model, with subsequent drug resistance assay. AGS (intestinal type) and Hs746T (diffuse type) gastric cancer cell lines were encapsulated in collagen beads with high cellular viability. AGS exhibited an aggregation pattern with expansive growth, whereas Hs746T showed single-cell-level infiltration. Importantly, in microtumor models, epithelial-mesenchymal transition (EMT) and metastatic genes were upregulated, whereas E-cadherin was downregulated. Expression of ß-catenin was decreased in drug-resistant cells, and chemosensitivity toward the anticancer drug (5-FU) was observed in microtumors. These results suggest that *in vitro* microtumor models may represent a biologically relevant platform for studying gastric cancer cell biology and tumorigenesis, and for accelerating the development of novel therapeutic targets.

Gastric cancer (GC) is an aggressive malignant tumor with high incidence and mortality in worldwide despite recent advances in anti-cancer drugs^1^. Lauren’s classification distinguishes the following two types of gastric cancer according to the morphological aspects of the tumor: (1) intestinal type (with a tubular and mucinous adenocarcinoma) and (2) diffuse type (poorly differentiated carcinoma). These morphological differences indicate distinct clinical phenotypes based on the presence of different molecular mechanisms^2^,^3^,^4^. Most cancer cells in the diffuse type are scattered and accompanied by marked stromal reactions^2^,^3^. Diffuse type GC is usually associated with higher mortality^5^.

To most effectively study the tumorigenesis and test the anti-cancer drugs, preclinical tumor models are required to reflect *in vivo* tumor microenvironments^6^,^7^,^8^. To date, great improvements in the construction of cancer models have been made using *in vitro* 3D physiologically relevant culture systems^9^,^10^,^11^,^12^,^13^. In general, *in vitro* 3D microtumor models can represent cell-cell and cell-extracellular matrix (ECM) interactions that are natural characteristics of *in vivo* microenvironment^14^,^15^,^16^,^17^. In cancer biology, the ECM regulates the physical and biochemical properties of the tumor microenvironment, which modulates cancer cell polarity, migration and signaling^18^,^19^,^20^. Stromal components of the ECM influence the biological behavior of tumor cells, especially ECM might impact more on diffuse type gastric cancer since it is existed in tumor environment with more abundant ECM compared to intestinal. Moreover, the ECM microenvironment play a primary role in the control of epithelial–mesenchymal transition (EMT) responses, which is a physiological process in which epithelial cells acquire motile and invasive characteristics^21^. In particular, the ECM in gastric cancer has been associated with increased mortality in patients^22^. In addition, EMT is an important event in the gastric cancer invasion–metastasis cascade whereby epithelial cells lose polarity along with cell–cell adhesion^23^,^24^.

Currently, many researchers are promoting 3D platforms for studying the interactions of tumor cells with the microenvironment and identifying the key factors that regulate the mode of migration and EMT responses^25^,^26^. However, the representative histological subtypes of gastric cancer (intestinal and diffuse) have not yet been well characterized in 3D models. As a valuable 3D model for the high-throughput evaluation of drugs, cell-laden ECM hydrogels can provide a more comprehensive assessment of tumor responses to therapeutic strategies, and enable the study of ECM-related tumor microenvironments.

Here, we demonstrated a droplet-based microtumor model to assess cell-ECM interactions and drug resistances of different types of gastric cancer, using the AGS (intestinal type) and Hs746T (diffuse type) cell lines. With this model, we performed a systematic comparison between 2D and 3D system in cultured cell characteristics and functional assessment. Type 1 collagen, which consisted of fibrous proteins and one of abundant ECM components in gastric tissue. By using a microfluidic-based droplet formation, we obtained well-controlled cell-encapsulated ECM microbeads. In both cell types, the expression of pro-metastatic genes, such as those involved with EMT, were upregulated in our model compared with 2D monolayer culture. Moreover, we confirmed that the drug resistance-related molecules were significantly up-regulated in 3D microtumor model of both cancer types; thus, the chemosensitivity against the anticancer drug, 5-fluorouracil (5-FU) is correlated with the expression of drug-resistance genes and proteins.

## Results

### Generation of cell-laden collagen bead

**Figure 1:**
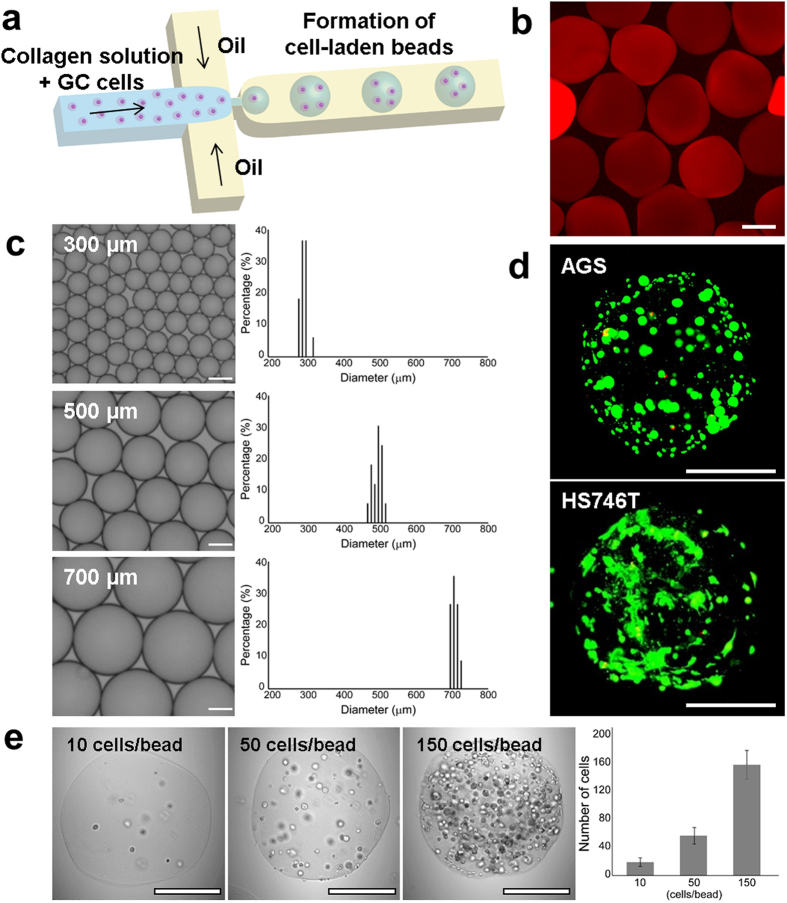
Formation of gastric microtumors. (**a**) Schematic illustration of forming gastric microtumors in microfluidic device. Cells encapsulated in collagen beads spontaneously form a size-controlled microtumors. (**b**) Immunofluorescence staining for type 1 collagen of collagen beads (red). (**c**) Image of generated microbeads and graphs by size distribution. By changing the flow rate of continuous and disperse phase, the size of microbeads controlled (n = 30–40). (**d**) Live/Dead staining of gastric cancer cells (AGS and Hs746T) in collagen microbead after 7 days. Live and dead cells were labelled with Calcein AM (green) and EthD-1 (red), respectively. (**e**) Images and graph of well-controlled cell concentration in microbead, 10, 50, and 150 cells per bead (n = 30–40). All scale bars: 300 μm.

Figure 1a shows a schematic illustration of the design of the microfluidic device. The polydimethylsiloxane (PDMS)-based microfluidic device is functionally composed of an aqueous channel with gastric cancer cells, collagen solution and oil. The dimension of microfluidic device is described in Supplementary Figure S1. To generate a biocompatible procedure for forming cell-laden microdroplets, we utilized a fluorinated oil (HFE7500) as oil phase, which has high oxygen permeability to ensure an adequate supply of oxygen during collagen gelation^27^. The microdroplets spontaneously formed at a cross junction due to their different interfacial properties^28^. First, 4 mg/ml collagen type 1 with GC cells and oil were injected into the device. Microdroplets with tunable and uniform sizes were generated using this system. By changing the flow rate of oil and aqueous phase, the diameter of the microdroplets ranged from 298.1 ± 9.3 μm to 715 ± 8.9 μm (Fig. 1c and Supplementary Movies 1,2,3). By modulating the flow rate of aqueous and oil phases, we were able to control the size of microbeads (Fig. 1c). To optimize the culture condition in collagen beads, we set the size to 500-μm, a size that could enable the transport of sufficient oxygen, nutrients and metabolites to the cell (Supplementary Figure S3). Under these conditions, hundreds of spherical microbeads containing GC cells were obtained. The generated collagen microbeads are identified immunofluorescence staining by anti-type 1 collagen (Fig. 1b), and microarchitecture of collagen hydrogels is observed by scanning electron microscopy (SEM) (Supplementary Figure S2). To optimize the cell concentration for a long-term 3D culture of a maximum of 10 days, the density of the cells in the ECM beads was controlled by modulating the initial cell concentration, which ranged from 10 to 150 cells per bead (Fig. 1e and Supplementary Figure S4). After gelation, the oil layer was removed to avoid the issue of mass transport impairment of nutrients and metabolites for cell viability. Initially, the cells were randomly distributed and evenly scattered in each collagen bead. During cell growth, the sparse cells became denser and more compact (Supplementary Figure S5). Live/dead cell staining assays showed that most of the GC cells were alive in the collagen bead after 7 days (Fig. 1d).

### Culture of gastric cancer cells in microbeads

**Figure 2:**
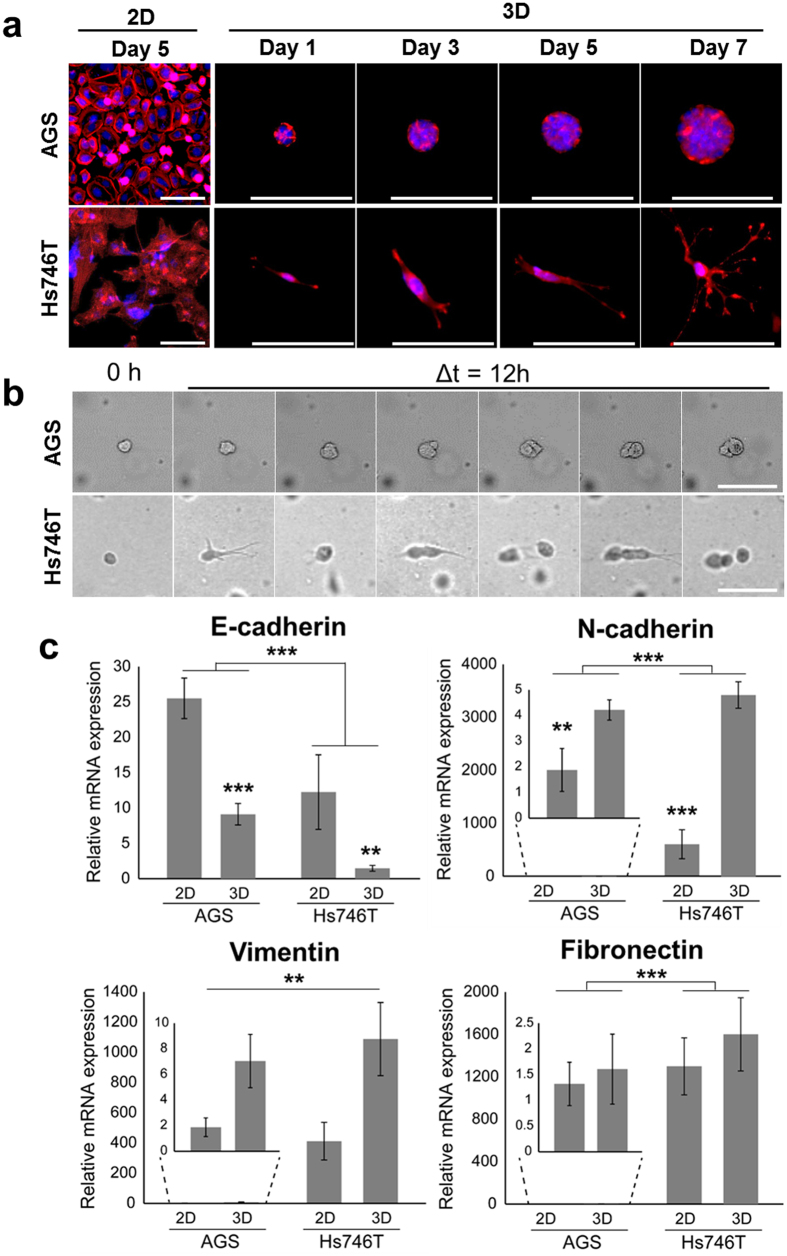
Cell growth in microtumor over time for the two types of gastric cancer. (**a**) Confocal images of 2D- and 3D-cultured AGS (top) and Hs746T (bottom) at different times. Immunofluorescence images show overlap of F-actin (red) and the nucleus (blue). Scale bars: 100 μm. (**b**) Sequences of images show the morphological features of gastric cancer cells in microtumors. Images were taken every 12 h. Intestinal-type cancer cells, AGS (top), present expansion growth forming cell aggregates, whereas diffuse-type cells, Hs746T (bottom), exhibit single-cell-level movement. Scale bar: 100 μm. (**c**) Differences in gene expression between two types of cell. Epithelial-mesenchymal transition (EMT)-related genes, E-cadherin, vimentin, fibronectin, and N-cadherin, are targeted (n = 4–5, **:p < 0.01, ***:p < 0.001).

Figure 2a shows the sequence of growth pattern for gastric microtumor formation at 1, 3, 5 and 7 days. To investigate the cell morphology in 2D and 3D microtumors, F-actin was stained with TRITC-phalloidin (red), and nucleus was stained with DAPI (blue). The two types of GC cells exhibited significantly different morphologies during cell growth (Fig. 2a). AGS cells aggregated and formed a spheroid, whereas Hs746T remained segregated with scattered structures. The size of individual spheres of AGS in beads is increased from 12.8 μm to 51.2 μm for 7 days. The diameter of spheroids in collagen beads was 51.2 ± 24.7 μm after 7 days. For Hs746T in beads, the length of cell bodies was approximately 41.3 μm to 149.2 μm. In a single-cell-level analysis, we observed that gastric cancer cells had different cell migration and invasive patterns depending on cell types (Fig. 2b and Supplementary Movies 4 and 5). AGS is spontaneously formed spheroid by re-positioning daughter cells rather than by active migratory mobility. In contrast, Hs746T cells showed unique elongated dendritic morphology in collagen microbeads and exhibited a single-cell-level invasion and exhibited F-actin rich cellular protrusions (i.e., invadopodia).

### Analysis of epithelial-mesenchymal transition (EMT)

**Figure 3:**
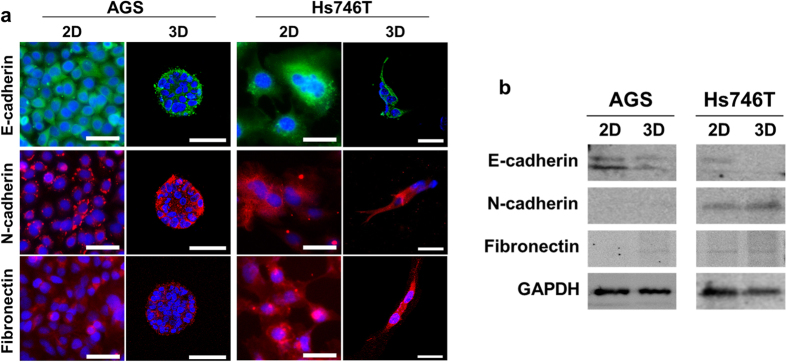
Expression of EMT-related proteins. (**a**) Representative immunofluorescence staining images of 2D gastric cancer cells, as well as 500-μm collagen bead-based 3D cultures (7 days). Cross-sections were fluorescently labeled with antibodies targeting the epithelial marker, E-cadherin (green), and the mesenchymal marker, N-cadherin, fibronectin (red). Nuclei were counterstained with DAPI (blue). Scale bars, 50 μm. (**b**) Immunoblot assays of gastric cancer cells in 2D and 3D after culturing for 7 days. Band signals were normalized to the GAPDH loading control.

Next, we analyzed the EMT-related gene expression of epithelial (E-cadherin) and mesenchymal (N-cadherin, vimentin, and fibronectin) markers. For the epithelial marker E-cadherin, the relative mRNA expression levels were significantly (2.7- and 8.3-fold) higher in the adherent 2D platform than in the 3D platform of AGS and Hs746T, respectively. In contrast, 3D cultured cells showed increases in mesenchymal markers compared with the 2D monolayer culture (Fig. 2c). In addition, a significant increase of mesenchymal markers in Hs746T was identified. For mesenchymal markers, the relative mRNA expression levels of N-cadherin were 2.2- and 5.7-fold higher in the 3D models of AGS and Hs746T, respectively. The relative mRNA expression of vimentin was 3.7- and 2.6-fold higher in the 3D models of AGS and Hs746T. In the case of fibronectin, there were no major differences between 2D and 3D cultures, but there was a notable difference between the different cell lines, AGS and Hs746T. Immunofluorescence staining of AGS in collagen beads showed highly polarized spheroids, as indicated by E-cadherin (Fig. 3a). Western blotting was also performed to quantify EMT-related proteins. Figure 3b shows the correlations between the relative mRNA (Fig. 2c) and protein expressions. Although the results showed both intestinal and diffuse types, GC cells acquired mesenchymal traits at the molecular level, mRNA in 3D. It is also explained by mRNA profiling of matrix metalloprotease (MMP) in 2D and 3D microtumors also supported these findings (Supplementary Figure S6).

### Analysis of drug resistance

**Figure 4:**
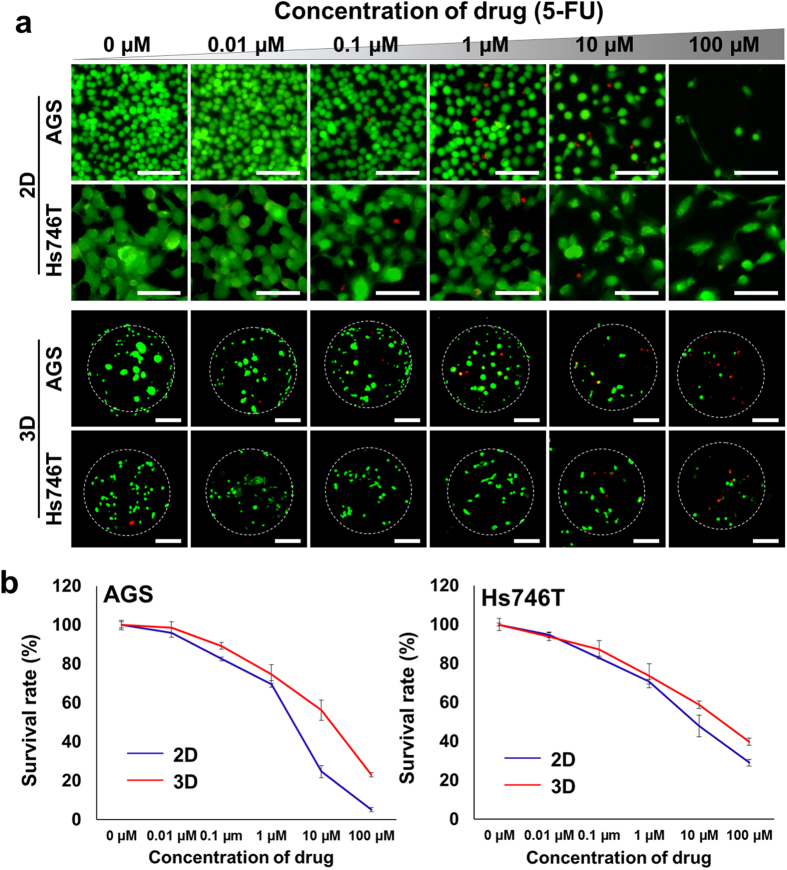
Evaluation of chemosensitivity in microtumor. (**a**) Live/dead staining of gastric cancer cells encapsulated in collagen beads after two days treatment at several 5-FU concentrations. Live and dead cells were stained with calcein AM (green) and EthD-1 (red) respectively. Scale bars: 50 μm. (**b**) Survival rate of AGS (right) and Hs746T (left) using WST1 proliferation assay (n = 3, blue line: 2D control, red line: 3D microtumor).

We evaluated drug resistance in 3D compared with 2D with two different gastric cancer cell-lines by culturing with standard gastric cancer chemotherapeutic agents, 5-FU, which is a typical apoptosis-inducing agent. We performed a Live/dead cell staining and WST-1 proliferation assay to determine changes in cell viability in response to drug treatment (Fig. 4 and Supplementary Fig. S7). To evaluate the half maximal inhibitory concentration (IC_50_) of gastric cancer cells, we treated cells with 5-FU at different concentrations, ranging from 0 to 100 μM, for 5 days after 1 day culture. Live/dead staining was conducted in 2D and 3D microtumor depending on the drug concentration, ranged from 0 to 100 μM. As shown in Fig. 4b, the drug concentration used was related to the change in cell viability. Although the survival rate was not substantially different between 2D and 3D at low concentrations (0.01 μM to 0.1 μM) of the drug, there was a difference in survival rate at high concentrations (10 μM to 100 μM). The 3D microtumor model exhibited more resistance to 5-FU at high concentrations of the drug. The IC_50_ of the 2D cultured AGS was 2-fold lower than that of the 3D microtumors, whereas the IC_50_ of Hs746T was approximately 10 μM and was similar in 2D and 3D (Supplementary Figure S7). Next, we treated cells with 5-FU for 7 days based on the IC_50_ (5 μM) of AGS in 2D model (Supplementary Figure S8). AGS cells showed 68.8% and 90.6% survival upon drug treatment in 2D and 3D cultures. On the other hand, Hs746T cells showed 87.2% and 94.7% survival after drug treatment, compared with the untreated control. A slight increase in survival of up to 7.5% was shown in the 3D microtumors compared with the 2D culture.

### Analysis of ß-catenin

**Figure 5:**
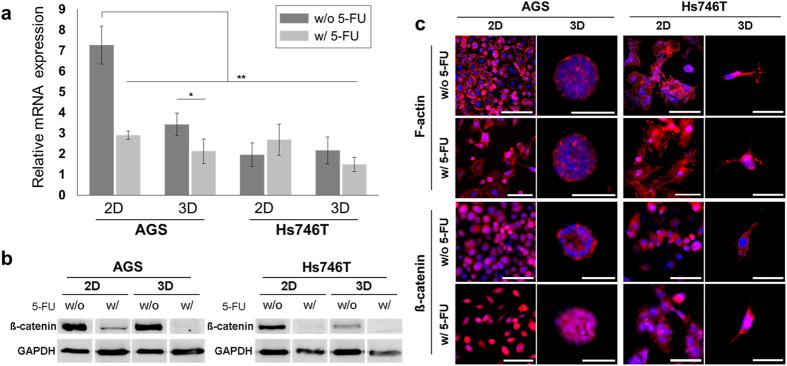
Expression of ß-catenin in gastric microtumors. (**a**) mRNA analyses of ß-catenin in 2D and 3D cultures of AGS (left) or Hs746T (right), with and without 5-fluorouracil (5-FU)] treatment. N = 4–5. **: p < 0.01, *: p < 0.05. (**b**) Western blot analysis of ß-catenin expression in AGS (left) and Hs746T (right) cells in 2D and 3D cultures with and without drug treatment. Band signals were normalized to the GAPDH loading control. (**c**) Cell morphology following 5-FU treatment in 2D and 3D cultures. Colored images represent the merge of F-actin (red) and nucleus (blue) staining (top). For immunofluorescence staining images of ß-catenin following 5-FU treatment, the colored images represent the merge of ß-catenin (red) and nucleus (blue) staining (bottom). Scale bars, 100 μm.

To investigate drug resistance in the gastric cancer model, we targeted ß-catenin for potential predictive biomarkers for further validation. Also, we performed cytoskeleton and ß-catenin staining to determine changes in cell morphology in response to drug treatment (Fig. 5). Subsequently, we investigated the correlation between ß-catenin expression and chemosensitivity. As shown by RT-qPCR and western blotting (Fig. 5), the relative mRNA expression of ß-catenin in AGS was significantly decreased approximately 4.3-fold in the 3D model, whereas the relative mRNA expression of ß-catenin in Hs746T exhibited no difference between 2D and 3D. In particular, the mRNA expression of ß-catenin was significantly decreased about 3-fold in Hs746T, compared AGS in 2D. These data indicate that reduced ß-catenin levels are associated with increased drug resistance in gastric cancer cells (Fig. 5a,b). Cells surviving drug treatment exhibited decreased ß-catenin expression in cell lines cultured in 2D and 3D. Additionally, expression of ß-catenin translocated to the nucleus in cells surviving 5-FU treatment (Fig. 5c).

## Discussion

The approach of a microfluidic-based cell-laden scaffold has several advantages as a cell-culture model. For instance, microfluidic-based droplet formation allows us to precisely control the size of confinement as well as the density of cell seeding by adjusting the experimental parameters, such as flow velocity and concentration of cell suspension. In fact, both microbead size and cell density could affect the cell viability as well as the total concentration of cell-released molecules due to limited diffusion. Therefore, identical and distinct properties could give rise to more efficient and accurate formation of tumor aggregates in 3D ECM with temporal and spatial control, as compared to other conventional bulk 3D systems. Furthermore, the microfluidic-based scaffold formation enables the establishment of rapid and high-throughput fabrication of microbead-based 3D tumors with small sample volumes. Therefore, we believe that a simple and well-characterized platform for the rapid formation of a 3D tumor model could improve the 3D culture system in high-throughput drug testing and screening.

Compared with a 2D monolayer culture, the characterization of the 3D culture indicated differences in cell activity, including morphology, proliferation, and gene and protein expression. Based on our observations, the morphologic differences between intestinal (AGS) and diffuse (Hs746T) GC cell types are exhibited in this model. AGS had an expansive growth pattern with formation of well-organized spheroids. Hs746T acquired a fibroblast-like, spindle-shape morphology and showed active single-cell movement (Fig. 2a,b). It means that the Hs747T cells form extended protrusion and ECM adhesion. The morphological differences between the cell types are consistent with the features of *in vivo* GC cells. Histologically, intestinal type GCs cells have been characterized by cohesive gland-like tubular structures with an expanding growth pattern. In contrast, diffuse type GC consists of fewer or no cohesive cells and grown diffusely spread in the gastric wall.

During GC development, the activation of EMT plays an important role in progression, invasion and metastasis^24^. EMT is a process by which epithelial cells are converted into mesenchymal cells in the gastric mucosa, and it involves significant phenotypic changes such as the loss of cell-cell adhesion, cell polarity and the acquisition of migratory and invasive properties^29^. Adhesion of tumor cells to ECM components is a pivotal step in developing peritoneal dissemination of intra-abdominal malignancy and is accompanied by the EMT. It is well-known that the diffuse type gastric cancer exhibits defective intercellular adhesion, primarily as a consequence of the loss of E-cadherin expression^30^. Moreover, the diffuse-type cancer exhibits altered expression of genes related to cell-matrix interactions and ECM components, whereas intestinal-type GC is represented by the enhancement of cell growth and maintenance of cell-cell interaction^24^.

As shown in Fig. 2, we observed the morphological changes in GC cells in the collagen beads. In particular, Hs746T cells had typical morphologic mesenchymal phenotypes. In addition to two morphologically different variants, diffuse-type and intestinal-type gastric cancer may also show differences in the genetic feature such as expression of the EMT regulators. The loss of functional E-cadherin in gastric cancer has been described in up to 50% of cases of diffuse-type gastric cancer, but not in the intestinal type^31^. In addition, the upregulation of mesenchymal markers has been observed in the diffuse type. These results suggest the existence of distinct carcinogenetic pathways for the intestinal and diffuse type carcinoma. Our RT-qPCR results confirmed that E-cadherin expression was significantly reduced, and expression of mesenchymal markers was upregulated in Hs747T when compared with the AGS (Fig. 2c). These findings indicate that the mode gastric microtumors reflect the characteristics of their *in vivo* gastric cancer counterparts, such as cell morphology and EMT-related gene expression.

Recently, EMT has been found to play a critical role in the cancer drug resistance, metastasis and poor prognosis of GC^32^. In this study, we determined that gastric cancer cells (AGS and Hs746T) undergo EMT and promoted cell migration and invasion through the β-catenin pathway. A variety of signalling pathways are related to the EMT induction, including tumor growth factor-β (TGF-β), nuclear factor-κB, Notch, RTK/Ras and Wnt/β-catenin^29^,^33^. In particular, ß-catenin signalling pathway plays an important role in EMT and drug resistance^32^. On the basis of the results, we speculated that EMT may be involved in the development of drug resistance that exhibit a negative correlation with the expression of ß-catenin.

In drug resistance tests, we determined the half maximal inhibitory concentration (IC_50_) against of 5-fluorouracil(5-FU), which is a commonly used chemotherapeutic agent for treating malignant GC^24^. Compared with AGS, Hs746T showed more malignancy and resistance to the 5-FU, with an approximately 2-fold of magnitude higher IC_50_. Drug resistance was increased in 3D microtumors compared with 2D, with significant differences at high concentration of drug (Fig. 4). Figure 4 represent that intestinal AGS cells acquire drug resistance when cultured in 3D while diffuse Hs746T cells maintain relatively similar characteristics. On the other hand, diffuse Hs746T cells did not undergo drastic changes in drug resistance; it could be speculated that diffuse type cells already exhibit cell-intrinsic drug resistance property. In addition, a substantial decrease in total ß-catenin levels in drug-resistant cells was observed, which likely resulted from the development of a mesenchymal phenotype associated with the loss of E-cadherin/β-catenin-associated adherent junctions. Subsequently, the lower expression of ß-catenin is associated with regulation of EMT for the progression of GC and drug resistance. Such a phenomenon has previously been shown to be involved in metastatic gastric cancer^34^. Indeed, the increased nuclear β-catenin level was observed by immunofluorescence staining (Fig. 5c) and was associated with increased drug resistance and decreased β-catenin expression. That is nuclear β-catenin is related high drug resistance and decreased expression of total β-catenin. Through β-catenin inhibitor, PNU-74654, we observed inhibition of nuclear transportation of β-catenin reduced drug resistance (Supplementary Figure S9).

Taken together, we demonstrated that distinct genotypic and phenotypic characteristics were associated with two different pathological variants of GC. According to the *in vivo*-like characteristics, we propose that gastric microtumors represent a physiologically relevant *in vitro* model to study EMT-mediated drug resistance in gastric cancer. The differences in the pathological behaviors of both types are of practical significance in decision making about appropriate treatment options in GC.

In summary, this study reports that a microdroplet-based 3D gastric cancer model provides a valuable 3D tumor microenvironment. Using our *in vitro* 3D GC models, we investigated different cellular responses between pathological variants of GC. Furthermore, we confirmed that the expression levels of ß-catenin were correlated with drug resistance. We believe that our microtumor platform could provide a better understanding of ECM-cell interactions and cancer invasion, and evaluate drug-resistant cancer in a controlled microenvironment.

## Methods

### Preparation of microfluidic devices

Microfluidic polydimethylsiloxane (PDMS) devices were fabricated by photolithography and soft lithography. The device design was drawn in AutoCAD to be used as photomask. The microfluidic device is functionally composed of an aqueous channel with gastric cancer cells, collagen solution and oil (Supplementary Figure S1). Dimension of device is described in detail in Supplementary Figure 1. Patterned silicon mold of 100 μm in height was prepared from SU-8 2100 (MicroChem) according to protocol. PDMS prepolymer and curing agent (Sylgard 184 Silicon Elastomer Kit, Dow Corning) were mixed at 10:1 ratio before poured on silicon mold. After curing at 70 °C for 2 hours, PDMS was peeled off from the mold before holes at inlets and outlets were punched. A flat PDMS sheet was bonded with the device after oxygen plasma treatment (Femto Science, Cute) for 30 s.

### Gastric cancer cell culture

The gastric cell lines, AGS and Hs746T, were obtained from Yonsei University. The gastric cancer cells were cultured in RPMI1640 media at 37 °C in a humidified incubator with 5% CO_2_. The culture dish is used size of 100 × 20 mm (SPL Life technology). The culture medium was supplemented with 10% fetal bovine serum (FBS, Welgene) and 1% penicillin/streptomycin (Welgene). AGS and Hs746T were subcultured by trypsin/EDTA (0.25%, Welgene). The culture media were changed every 2~3 days.

### Generation of cell-laden collagen beads (microtumors)

Collagen type 1 (Rat tail, Corning) was diluted and neutralized to 4 mg/ml in ice bath to prevent gelation^35^. Collagen stays liquid at 4 °C but self-assembles into a gel at 37 °C. To generate a biocompatible procedure for forming cell-laden microdroplets, we utilized a fluorinated oil (HFE7500) as oil phase, which has high oxygen permeability to ensure an adequate supply of oxygen during collagen gelation. Collagen microdroplets formed at the cross-junction by flow focusing with a continuous stream of low-viscosity fluorocarbon oil, HFE7500 (3 M Novec, Singapore), loaded with 2 wt% surfactant, 008-FluoroSurfactant (Ran biotechnology, USA). During injection, the syringe filled with the collagen solution was covered with an ice bag to prevent gelation of collagen. Droplets were generated in a microfluidic channel using a syringe pump (70-4506INT; Harvard Apparatus, Holliston, MA, USA) and a Teflon tube (inner diameter, 0.02 inches; outer diameter, 0.06 inches). The microdroplets spontaneously formed at a cross junction due to their different interfacial properties. First, 4 mg/ml collagen type 1 with GC cells and oil were injected into the device. The optimal flow rates for the formation of stable 500-μm microbeads were 4 μL/min for a cell-laden ECM solution channel and 10 μL/min for an oil channel. The optimal flow rates for the formation of stable 300- and 700-μm microbeads were 5 μL/min for a cell-laden ECM solution channel and 13 and 20 μL/min for an oil channel, respectively. Droplets were collected externally in an Eppendorf tube at outlet part, and incubated at 37 °C incubator to make collagen beads. After 30 min at 37 °C, the microtumors were released using a droplet releasing agent (1H,1H,2H,2H-perfluoro-1-octanol) from Sigma-Aldrich^27^,^36^,^37^,^38^. In a sterilized tube, the droplet releasing agent and microtumors (variable volume) were added sequentially, and left at RT for 5 minutes. The released microtumors in aqueous phase was then retrieved. Retrieved microtumors are cultured in RPMI1640 media at 37 °C in a humidified incubator with 5% CO_2_ for 7 days, and the medium was changed every 2~3 days, same as 2D cultured.

### Cell viability assay

Cells cultured in 2D and 3D microbeads were suspended in PBS and mixed with Live/Dead viability/cytotoxicity kit (Life technology), consisting of Calcein AM (4 mM) and ethidium homodimer-1 (EthD-1, 2 mM). Live/Dead staining solution (2 μM Calcein AM and 4 μM EthD-1 in PBS) was introduced into 2D and 3D cultured gastric cancer cells for 20 min at room temperature. The viability was indicated by green (live) or red (dead) fluorescence labelled cells. Live and dead cells observed by measuring fluorescence signal using laser scanning confocal microscopy (Nikon).

### Immunofluorescent staining

Cultured cells in 2D and 3D were fixed with 4% paraformaldehyde solution (Biosesang, Korea), and permeabilized with 0.15% Triton-X100 (Sigma-Aldrich) in PBS. And then cells were blocked with 2% bovine serum albumin (BSA, Sigma-Aldrich) in PBS for 1 h at RT. After blocking, cancer cells in 2D and 3D were incubated with the antibody diluted in PBS with 1% BSA. F-actin and nucleus of cells was stained with TRITC-phalloidin (1:1000, Sigma-Aldrich) and DAPI (1:2000, Sigma-Aldrich) for 20 min at RT. For immunostaining of EMT and drug resistant-related proteins, cancer cells were incubated with anti-ß-catenin (1:200, Abcam), E-cadherin (1:100, Abcam), N-cadherin (1:150, Abcam), and Fibronectin (1:150, Abcam) overnight at 4 °C. Subsequently, TRITC-conjugated secondary antibody (dilution 1:500) and FITC-conjugated secondary antibody (dilution 1:500) was incubated for 3 hours at room temperature. Fluorescence images were acquired using laser scanning confocal microscopy (Nikon).

### Total RNA extraction from gastric cancer cells in 2D and 3D culture

The mRNA expression associated with EMT and drug resistance was analyzed by RT-qPCR. To obtain samples for reverse RT-qPCR, cells in 2D and 3D were cultured on a cell culture dish (100 × 20 mm; SPL Life Sciences, Pocheon, Korea). To analyze cell proliferation and drug resistance, cells were cultured in 96-well plates. For the harvesting of cells and RNA extraction, 2D-cultured cells were scraped off the dish bottom using a cell scraper. 3D microtumors were carefully collected using a pipette. Then, 2D- and 3D-cultured samples were homogenized by hand using a tissue grinder on ice. Isol RNA lysis reagent (5 Prime, Hilden, Germany) was used to extract total RNA from cells in accordance with the manufacturer’s instructions. Samples were mixed with this reagent, followed by phase separation using chloroform. After collection of the aqueous phase, RNA was precipitated by treatment with isopropyl alcohol and glycogen for 1 h at −20 °C. Extracted RNA was collected and washed with 70% ethanol three times, followed by dehydration. Dried RNA was dissolved in a suitable amount of diethylpyrocarbonate water. The concentration of total RNA was determined by measuring the absorbance at 260 nm using a spectrophotometer. Only RNA samples with A260/A280 ≥ 1.8 were used. Samples were stored at −20 °C until future use.

### Quantitative real time reverse transcription-polymerase chain reaction (RT-qPCR) analysis

Total RNA (1 μg) was reverse-transcribed into cDNA by the iScript cDNA synthesis kit (Bio-Rad, Hercules, CA, USA). RT-qPCR were performed in a thermal cycler using SYBR Green Real-time PCR Master Mix (Toyobo, Osaka, Japan). RT-qPCRs were conducted using primers for ß-catenin; Forward: 3′-GCT GAT TTG ATG GAT GGA GTT GG-5′, Reverse: 3′-CTA CTT GTT CTT GAG TGA A-5′, N-cadherin; Forward: 3′-TTT GAG GGC ACA TGC AGT AG-5′, Reverse: 3′-ACT GTC CCA TTC CAA ACC TG-5′, E-cadherin; Forward: 3′-GTG ACT GAT GCT GAT GCC CCC AAT ACC-5′, Reverse: 3′-GAC GCA GAA TCA GAA TTA GGA AAG CAA G-5′, Vimentin; Forward: 3′-GAA ATT GCA GGA GGA GAT GC-5’, Reverse: 3′-CGC ATT GTC AAC ATC CTG TC-5′, and Fibronectin; Forward: 3′-ATG AGC TGC ACA TGT CTT GG-5′, Reverse: 3′-TGG CAC CGA GAA TTC CTT C-5′. The expression levels were normalized to those of GAPDH; Forward: 3′-GTA TGA CAA CAG CCT CAA GAT-5′, Reverse: 3′-AGT CCT TCC ACG ATA CCA AA-5′. Quantification was based on the expression of the target genes relative to that of the housekeeping gene GAPDH as a reference (endogenous control). The normalized value was then expressed as the ratio relative to that in the 2D monolayer group.

### Western blot

Cells were lysed in 1X RIPA buffer (Sigma-Aldrich) supplemented with Halt™ Protease and Phosphatase Inhibitor Cocktail (ThermoFisher Scientific). The protein concentration was determined by Bradford method with BSA (Sigma-Aldrich) as the standard. Equal concentration of protein extract were subjected to 12% SDS-PAGE and transferred to PVDF membrane. Blots were labelled with the primary antibodies, ß-catenin (1:1000, Abcam), and GAPDH (1:2000, Sigma). GAPDH was used as a loading control. And following day, HRP-conjugated secondary antibody (1:5000, Sigma-Aldrich) was added to the blot for 3 hours at RT. Protein expression was detected using ECL western blotting detection reagent (Bio-Rad, Hercules, CA, USA) and membranes were imaged using an imaging system (ImageQuant LAS 4000 mini, GE Healthcare).

### Drug resistance experiments

Both AGS and Hs746T grown on culture dish and in collagen bead for 5days were treated with 5-FU in various concentrations. 5-FU were obtained from Yonsei University. To analyze drug resistance, we used 1 × 10^3^ cells/well in 2D culture and approximately 20 beads/well in 3D microtumor culture (50 cells per collagen bead). After 5 days of incubation, surviving cells were examined by the WST-1 assay (Roche Diagnostics, Mannheim, Germany). In all, 1 × 10^3^ cells were seeded into 96-well plates, and allowed to adhere to the dish or ECM for 24 h, at which point 0.01 μM to 100 μM 5-FU was administered. WST-1 solution was added to the cells, incubated at 37 °C for 2 h, and the signal measured using XMark microplate spectrophotometer (Bio-Rad, Hercules, CA, USA). The survival rate at different 5-FU concentrations were normalized to the measured value at 0 μM 5-FU.

### Statistical analysis

For all statistical analysis, the normalized values from independent experiments were averaged and expressed as mean ± standard error (SE). Data sets were statistically analysed for significance using two-tailed Student’s *t*-tests. In graph of Figs 2, 5 and S6, p-values are denoted with either asterisks (*) as follows: *:significant at p < 0.05, **:significant at p < 0.01, and ***:significant at p < 0.001.

## Additional Information

**How to cite this article**: Jang, M. *et al*. Droplet-based microtumor model to assess cell-ECM interactions and drug resistance of gastric cancer cells. *Sci. Rep.* **7**, 41541; doi: 10.1038/srep41541 (2017).

**Publisher's note:** Springer Nature remains neutral with regard to jurisdictional claims in published maps and institutional affiliations.

## Supplementary Material

Supplementary Information
